# Structural variations causing inherited peripheral neuropathies: A paradigm for understanding genomic organization, chromatin interactions, and gene dysregulation

**DOI:** 10.1002/mgg3.390

**Published:** 2018-03-23

**Authors:** Anthony N. Cutrupi, Megan H. Brewer, Garth A. Nicholson, Marina L. Kennerson

**Affiliations:** ^1^ Northcott Neuroscience Laboratory ANZAC Research Institute Sydney NSW Australia; ^2^ Sydney Medical School University of Sydney Sydney NSW Australia; ^3^ Molecular Medicine Laboratory Concord Hospital Sydney NSW Australia

**Keywords:** gene dysregulation, inherited peripheral neuropathies, structural variation, topological associated domains

## Abstract

Inherited peripheral neuropathies (IPNs) are a clinically and genetically heterogeneous group of diseases affecting the motor and sensory peripheral nerves. IPNs have benefited from gene discovery and genetic diagnosis using next‐generation sequencing with over 80 causative genes available for testing. Despite this success, up to 50% of cases remain genetically unsolved. In the absence of protein coding mutations, noncoding DNA or structural variation (SV) mutations are a possible explanation. The most common IPN, Charcot‐Marie‐Tooth neuropathy type 1A (CMT1A), is caused by a 1.5 Mb duplication causing trisomy of the dosage sensitive gene *PMP22*. Using genome sequencing, we recently identified two large genomic rearrangements causing IPN subtypes X‐linked CMT (CMTX3) and distal hereditary motor neuropathy (DHMN1), thereby expanding the spectrum of SV mutations causing IPN. Understanding how newly discovered SVs can cause IPN may serve as a useful paradigm to examine the role of topologically associated domains (TADs), chromatin interactions, and gene dysregulation in disease. This review will describe the growing role of SV in the pathogenesis of IPN and the importance of considering this type of mutation in Mendelian diseases where protein coding mutations cannot be identified.

## 1 INTRODUCTION

Inherited peripheral neuropathies (IPNs) are a group of diseases causing length‐dependent axonal degeneration of the peripheral motor and/or sensory nerves resulting in chronic disability. They are among the most common inherited neuromuscular diseases affecting approximately 1 in 2,500 people (Skre, 1974). IPNs are classified into one of three subtypes based on the pathology predominantly affecting the motor nerves (hereditary motor neuropathy—HMN), sensory nerves (hereditary sensory and autonomic neuropathy—HSAN) or both motor and sensory nerves (Charcot‐Marie‐Tooth neuropathy—CMT) (Baets & Timmerman, 2011). Over 1,000 mutations in more than 80 genes have been associated with IPN subtypes (Timmerman, Strickland, & Zuchner, 2014). However, despite these discoveries, numerous whole‐exome sequencing studies to screen IPN cohorts have shown 18%–50% of cases (or higher) remain unsolved creating a significant burden for IPN diagnosis (Drew et al., 2015; Hartley et al., 2017; Lupo et al., 2016; Schabhuttl et al., 2014). In these cases where exome sequencing has failed to identify mutations in specific genes, it is likely a proportion of the neuropathy cases may be due to noncoding DNA or structural variation (SV) mutations. We have recently identified novel SV mutations causing two forms of IPN – X‐Linked Charcot‐Marie‐Tooth neuropathy (CMTX3) (Brewer et al., 2016) and distal hereditary motor neuropathy (DHMN1) (Drew, Cutrupi, Brewer, Nicholson, & Kennerson, 2016). Our findings have added to the spectrum of mutations causing IPN and highlight the importance of interrogating the noncoding genome for SV mutations for IPN families that have been excluded for genome wide protein coding mutations. We also propose that the CMTX3 and DHMN1 disease causing genomic rearrangements may provide a suitable paradigm for studying the growing area of 3D genomic organization and its underlying role in mechanisms of gene dysregulation.

## 2 STRUCTURAL VARIATION

Genetic research in IPNs has been pioneering in the discovery of genomic disorders. Perhaps most importantly, it has expanded our understanding of the effects of copy number variation SVs in disease pathogenesis. Advancement in sequencing and genomic technologies including high‐throughput massively parallel sequencing, and array‐based techniques such as array comparative genomic hybridization (aCGH) has led to the development of diverse approaches for SV discovery as well as studying the functional and phenotypic consequences of SV rearrangements (Hoyle, Isfort, Roggenbuck, & Arnold, 2015; Hurles, Dermitzakis, & Tyler‐Smith, 2008; Weischenfeldt, Symmons, Spitz, & Korbel, 2013).

Structural variation is a broad term that encompasses genomic rearrangements that disrupt chromosomal organization and architecture. There are many types of structural variation including duplications and deletions (also referred to as copy number variation [CNV]), insertions, inversions, and translocations. These have been reviewed in detail elsewhere (Alkan, Coe, & Eichler, 2011; Guan & Sung, 2016; Stankiewicz & Lupski, 2010). The size of SV DNA rearrangements can range from thousands to millions of base pairs (Gu, Zhang, & Lupski, 2008). SV can also be complex. A simple SV event may be part of a more complex rearrangement involving 2 or more types of SV. Such examples include translocation insertions (Antonarakis, Kazazian, & Tuddenham, 1995; Brewer et al., 2016), translocation deletions (Barber, Ford, Harris, Harrison, & Moorman, 2004), and inversion duplications (Kostiner, Nguyen, Cox, & Cotter, 2002). The scale and complexity of SV can therefore cause major re‐organization of the regulatory landscape of genomic loci. It accounts for a considerable proportion of genetic variability, phenotypic diversity, and human disease (Feuk, Carson, & Scherer, 2006; Guan & Sung, 2016; Redon et al., 2006; Weischenfeldt et al., 2013) with SVs having more heritable differences between individuals than SNPs (Baker, 2012; Weischenfeldt et al., 2013). Reference to structural variation in both the scientific literature and in curated databases such as the Database of Genomic Variants (DGV‐ http://dgv.tcag.ca/dgv/app/home) has grown significantly (Baker, 2012; J. R. Lupski et al., 1991). SV represents an excellent candidate for disease causing mutations for IPN, especially when genome wide protein coding point mutations have been excluded in unsolved families.

### 2.1 Known structural variation causing IPN

The most common IPN subtype, CMT1A is caused by a 1.5‐Mb tandem duplication of chromosome 17p11.2 (Lupski et al., 1991) which results in trisomy of the gene encoding peripheral myelin protein 22 (*PMP22,* OMIM:*106907) (Lupski et al., 1992; Patel et al., 1992; Timmerman et al., 1992; Valentijn et al., 1992). The CMT1A duplication represents the first and most common IPN mutation (Inoue et al., 2001), accounting for approximately 50% of all CMT cases (Katona et al., 2009). Reciprocal deletion of the same chromosome 17p11.2 region causes hereditary neuropathy with liability to pressure palsies (HNPP) which results in a mild, episodic peripheral neuropathy (Chance et al., 1993). At the time of reporting the CMT1A duplication/HNPP deletion, this represented a seminal discovery for structural variation (CNV) causing IPN and demonstrated the sensitivity of nervous tissue to gene dosage changes due to the gain or loss of a copy of the *PMP22* gene.

Atypical genomic rearrangements have also been identified for the CMT1A/HNPP locus and other IPN loci (Table 1). For CMT1A these include duplications that differ in size to the 1.5 Mb genomic rearrangement (Valentijn et al., 1993) and small exonic deletions involving all or part of *PMP22* gene coding exons (Zhang et al., 2010). Two novel duplications that exclude the *PMP22* coding region have also been identified in cases of CMT1A (Weterman et al., 2010; Zhang et al., 2010). Weterman et al., reported a 186 kb duplication located upstream of the *PMP22* gene in six unrelated families (Weterman et al., 2010). Work by Zhang et al., examined the contribution of nonrecurrent genomic rearrangements to CMT1A/HNPP and confirmed the previously reported 186 kb duplication in 2 of 21 subjects. The study also identified a novel 194 kb duplication upstream of the *PMP22* gene in one patient (Zhang et al., 2010). Both the 186 kb and 194 kb duplication mutations shared a common overlapping region of approximately 168 kb (Zhang et al., 2010). This region has since been shown to contain several putative enhancers of the *PMP22* gene (Jones et al., 2011, 2012) which when duplicated cause CMT1A. Cases describing whole or partial gene duplications or deletions at other CMT loci include *MPZ* (OMIM:*159440) (Hoyer, Braathen, Eek, Skjelbred, & Russell, 2011; Maeda et al., 2012), *MFN2* (OMIM:*608507) (Carr et al., 2015), *GJB1* (OMIM:*304040) (Ainsworth, Bolton, Murphy, Stuart, & Hahn, 1998; Lin et al., 1999; Nakagawa et al., 2001), and *NDRG1* (OMIM:*605262) (Okamoto et al., 2014). Apart from these reports, however, CNV disrupting other IPN loci appear to be rare. Three independent studies used aCGH to examine the contribution of CNV to IPN and all concluded that apart from the CMT1A duplication/HNPP deletion, CNV as a disease mechanism in IPN is rare (Hoyer et al., 2015; Huang et al., 2010; Pehlivan et al., 2016).

**Table 1:** Structural variations reported for IPN loci

References	Phenotype	Description	Approximate Size (kb)	Mechanism	HGVS/ISCN (GRCh38/hg38)
Lupski et al., (1991)	CMT1A	Duplication involving *PMP22*	1,500	Gene dosage of *PMP22*	Chr17:g.(14170534_14194724)_(15567585‐15591587)dup
Valentijn et al., (1993)	CMT1A	Duplication involving *PMP22*	450	Gene dosage of *PMP22*	Undetermined
Zhang et al., (2010)	CMT1A	Duplication of sequences upstream of *PMP22*	194	Dysregulation of *PMP22* gene expression	Chr17:g.(15467580_15467581)ins(15274153_15467575)
Weterman et al., (2010)	CMT1A	Duplication of sequences upstream of *PMP22*	186	Dysregulation of *PMP22* gene expression	Chr17:g.(15485748_15485749)ins(15299622‐15485747)
Zhang et al., (2010)	CMT1A	Duplication involving *PMP22*	412	Gene dosage of *PMP22*	Chr17:g.(15624595_15624596)ins(15213043‐15624585)
Zhang et al., (2010)	CMT1A	Deletion involving *PMP22*	536	Gene dosage of *PMP22*	Chr17:g.14709310_15245549del
Zhang et al., (2010)	CMT1A	Complex rearrangement involving *PMP22*	3,400	Gene dosage of *PMP22*	Undetermined
Zhang et al., (2010)	CMT1A	Complex rearrangement involving *PMP22*	1,294	Gene dosage of *PMP22*	Undetermined
Chance et al., (1993)	HNPP	Deletion involving *PMP22*	24	Gene dosage of *PMP22*	Chr17:g.(14170534_14194724)_(15567585_15591587)del
Nadal et al., (2000)	HNPP	Translocation	Undetermined	Dysregulation of gene expression	t(16;17)(q12;p11.2)
Ainsworth et al., (1998)	CMTX1	Deletion involving *GJB1*	Undetermined	Gene dosage of *GJB1*	Undetermined
Rouger et al., (1997)	CMTX1	Complex rearrangement involving *GJB1*	Undetermined	Undetermined	Undetermined
Maeda et al., (2012)	CMT1B	Duplication involving *MPZ*	117	Gene Dosage of *MPZ*	Chr1:g.(161415803_161415804)ins(161298102_161415774)
Brewer et al., (2016)	CMTX3	Complex Insertion	78	Dysregulation of gene expression	ChrX:g.140420783_140420784ins[chr8:g.144542928_144620773;TTCCTTCCT]g.138490706_138490718inv
Drew et al., (2016)	DHMN1	Complex Insertion	1,350	Dysregulation of gene expression	Chr7:153636339_153637495delins[GGTGCGGGCTCCT;g.155856471_157199742inv; AGTATGGCTGTAAGTGACGTC
Zhang et al., (2010)	CMT1A	Deletion	17	Gene dosage of *PMP22*	Chr17:g.15234989_15252302delins[CAT]
Lin et al., (1999)	CMTX1	Deletion involving *GJB1*	1.5	Gene dosage of *GJB1*	ChrX:g.71306403_71307859del
Nakagawa et al., (2001)	CMTX1	Deletion involving *GJB1*	1.5	Gene dosage of *GJB1*	ChrX:g.71306977_71307427del
Hoyer et al., (2011)	CMT1B	Duplication involving *MPZ*	4	Gene Dosage of *MPZ*	Chr1:g.161304727_161308898dup
Okamoto et al., (2014)	CMT4D	Exonic Duplication involving *NDRG1*	6.25	Gene Dosage of *NDRG1*	Chr8:g.133252822_133259076dup
Carr et al., (2015)	CMT2A	Exonic Deletion involving *MFN2*	Undetermined	Gene Dosage of *MFN2*	Undetermined

Undetermined = insufficient information provided in published data.

Complex structural variations have been described that disrupt known IPN loci and include rearrangements for CMTX1 involving the entire coding region (Rouger et al., 1997), as well as a reciprocal translocation t(16;17)(q12;p11.2) causing HNPP (Nadal et al., 2000). The involvement and contribution of non‐CNV structural variations as a pathomechanism in IPN therefore remains largely understudied and poorly understood.

### 2.2 Discovery of two complex insertions expands the mutation spectrum of IPN

**Figure 1:**
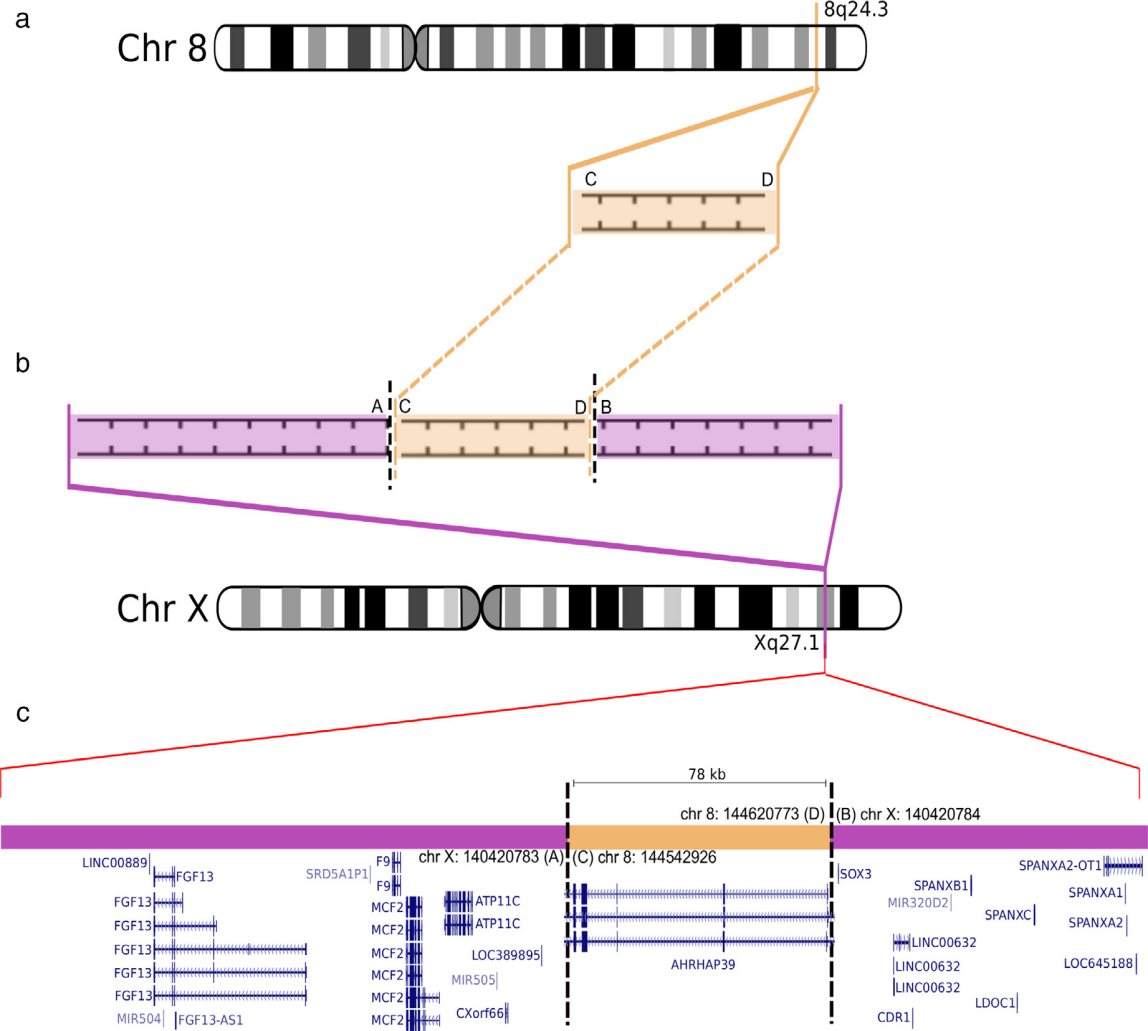
The CMTX3 complex insertion. Ideogram of chromosome 8 and chromosome X expanded to show (a) the region of chromosome 8q24.3 inserted into (b) the CMTX3 locus on chromosome X. Purple areas represent sequence contained within the CMTX3 locus. Orange areas represent the region of chromosome 8q23.4 inserted into the CMTX3 locus. Breakpoints are indicated by the vertical black broken lines. Letters A–D indicate sequences flanking the breakpoints. (c) Relative positions of the genes contained within the complex insertion and flanking the insertion site within the CMTX3 locus. Adapted from the gene track of UCSC Genome Browser (GRCh38/hg38) for the different genomic interval on chromosome Xq27.1 and chromosome 8q24.3

**Figure 2:**
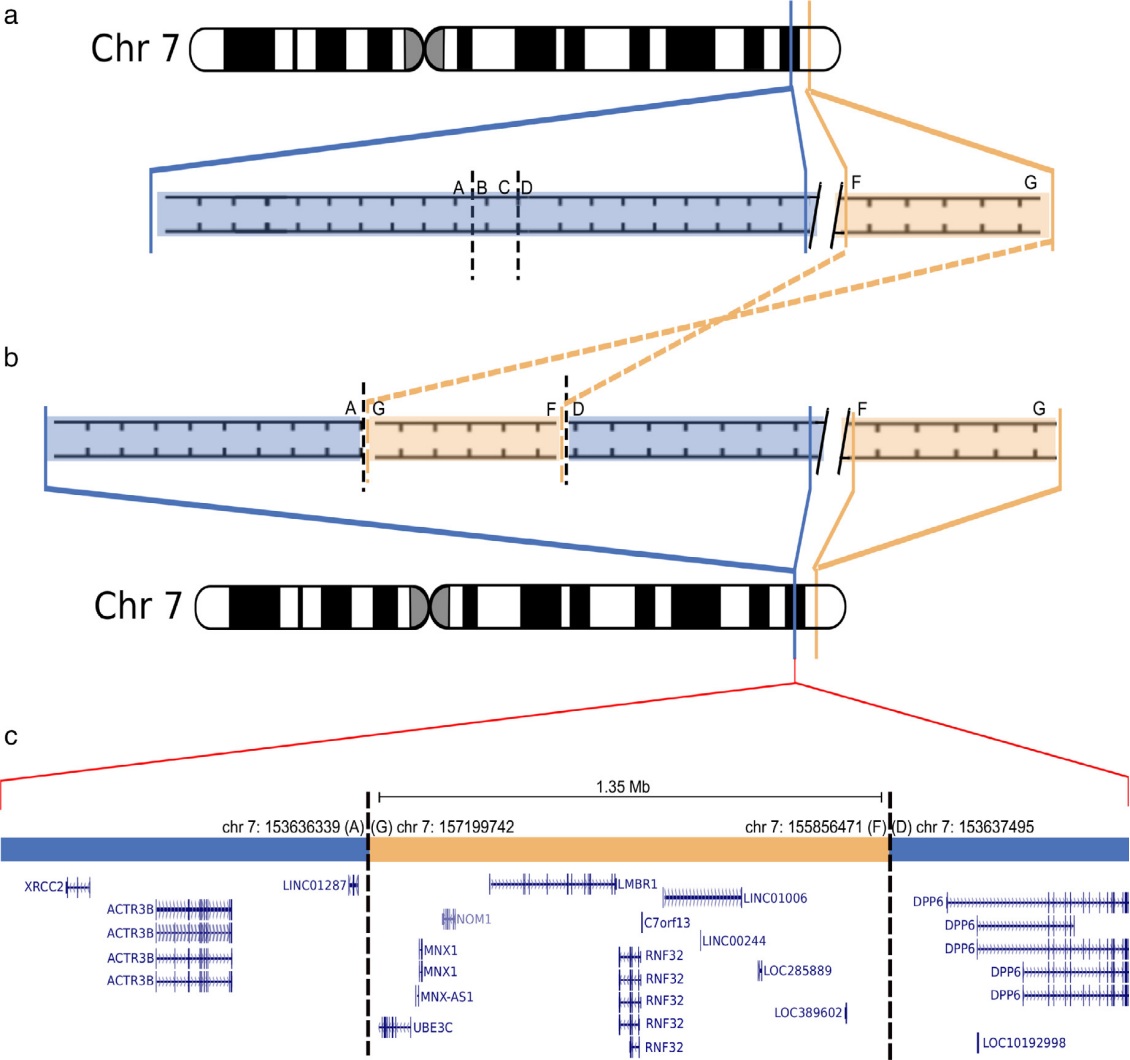
The DHMN1 complex insertion. Ideogram of chromosome 7 expanded to show (a) the normal and (b) DHMNI locus. Blue areas represent sequence within the DHMN1 locus. Orange areas represent sequence at chromosome 7q36.3 inserted into the DHMN1 locus. The chromosome 7q36.3 sequence is located 2.3 Mb distal to the DHMN1 locus. Breakpoints are indicated by the vertical black broken lines. Letters A–G indicate sequences flanking the breakpoints. The sequence FG from chromosome 7q36.3 has been inserted into the DHMN1 locus in inverted orientation. (c) Relative positions of the genes contained within the complex insertion and flanking the insertion site within the DHMN1 locus. Adapted from gene tracks of the UCSC Genome Browser (GRCh38/hg38) for the specific chromosome 7q36.3 and 7q36.2 genomic intervals

Our laboratory has recently identified two novel SVs as the underlying genetic causes of an X‐linked form of CMT (CMTX3, OMIM:%302802) (Brewer et al., 2016); and an autosomal dominant form of distal HMN (DHMN1, OMIM:%182960) (Drew et al., 2016). Linkage analyses in large families mapped the respective disease loci to chromosome Xq26.3‐q27.3 for CMTX3 (Huttner, Kennerson, Reddel, Radovanovic, & Nicholson, 2006) and chromosome 7q34‐q36.2 for DHMN1 (Gopinath, Blair, Kennerson, Jennifer, & Nicholson, 2007). Following identification of the respective disease loci, whole‐exome sequencing, CNV, and karyotype analyses failed to identify any candidate gene mutations for CMTX3 and DHMN1. Paired end whole‐genome sequencing (WGS) was then performed on multiple affected individuals from the CMTX3 and DHMN1 families and analyzed for split and discordant map reads. The analysis revealed a large complex insertion within the respective CMTX3 and DHMN1 disease locus. CMTX3 patients had a 78 kb duplication of chromosome 8q24.3 inserted into the CMTX3 locus at chromosome Xq27.1 (Figure 1). For DHMN1 patients, a 1.35 Mb duplication of chromosome 7q36.3 was inserted into the DHMN1 locus at chromosome 7q36.2 in the reverse orientation (Figure 2). Both SVs segregated with the disease in their respective families. The SVs were absent in the unaffected individuals sent for WGS, 1054 neurologically normal control chromosomes, and the DGV database.

**Figure 3:**
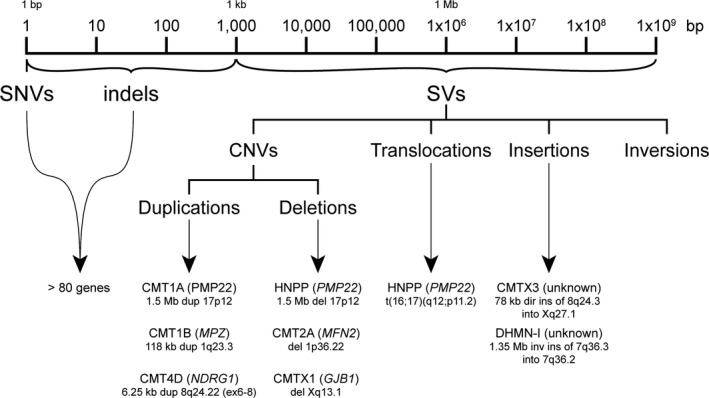
Structural Variation expands the mutation spectrum of IPN. Classes of genetic mutations and the size of the DNA re‐arrangements caused by structural variation. The two large complex insertions for CMTX3 and DHMN1 neuropathy highlight the growing role of structural variation in the pathogenicity of IPN

Our two SV discoveries expand the spectrum of mutations known to cause IPN (Figure 3) and raise some significant questions with respect to the biological impact of SV mutations in peripheral nerve and their role in gene dysregulation as a disease mechanism for IPN.

## 3 PATHOGENIC MECHANISMS OF SV MUTATIONS

Insights into the mechanisms that underpin the relationship between SV and disease pathogenesis have been garnered from studies on a range of diseases including Hemophillia A (reviewed in (Tuddenham et al., 1991)), Parkinson's Disease (Marongiu et al., 2007; Singleton et al., 2003), and Aniridia (Fantes et al., 1995; Fukushima et al., 1993; Simola, Knuutila, Kaitila, Pirkola, & Pohja, 1983). These studies have revealed that SVs can cause disease by several mechanisms including (i) physical disruption of the DNA structure of genes, (ii) the unmasking of recessive alleles or functional polymorphisms, (iii) alteration of gene dosage of dosage sensitive genes via copy number change, and (iv) disrupting/altering the spatiotemporal control of gene expression causing gene dysregulation/ectopic expression (Kleinjan & Lettice, 2008; Kleinjan & Van Heyningen, 2005; Spielmann & Mundlos, 2013). Given that the CMTX3 and DHMN1 SVs are complex insertions, we propose two possible mechanisms that could lead to the different neuropathy in our families: (i) altered gene dosage resulting from trisomy of the complex inserted regions; or (ii) transcriptional dysregulation of one or more genes mapping within the CMTX3 and DHMN1 disease loci and flanking regions.

The 78 kb CMTX3 duplication of chromosome 8q24.3 contains a partial transcript (exons 1–7) of the *ARHGAP39* (OMIM:*615880) gene which is encoded on the negative strand. The duplicated chromosome 8q24.3 sequence is inserted into an intergenic region of chromosome Xq27.1 with *LOC389895* and *SOX3* (OMIM:*313430) being the nearest flanking genes (Brewer et al., 2016) (Figure 1). The 1.35 Mb DHMN1 SV duplication of chromosome 7q36.3 involves five protein‐coding genes (*LOC389602*,* RNF32* (OMIM:*610241), *LMBR* (OMIM:*605522), *NOM1* (OMIM:*611269), *MNX1* (OMIM:*142994)), a partial transcript of the *UBE3C* (OMIM:*614454) gene (exons 1–9), three long noncoding RNAs (lncRNA; *LOC285889*,* LINC01006*,* MNX1*‐*AS1)* and several putative enhancers and promoters. The location of the insertion occurs within a gene desert of the DHMN1 locus between the proximal *ACTR3B* gene and the distal *DPP6* (OMIM:*126141) gene. The closest genomic element is a lncRNA *LINC01287* transcribed on the negative strand (Drew et al., 2016) (Figure 2).

### 3.1 Gene dosage

The CMT1A/HNPP duplication/deletion sets a precedent for a SV disease mechanism in IPN causing peripheral nerves to be sensitive to gene dosage changes. For both CMTX3 and DHMN1, trisomy of any gene transcripts (whole or partial) within the inserted DNA may result in gene dosage effects and produce neuropathy. For CMTX3, differential expression of *ARHGAP39* in lymphoblasts using qPCR was not observed between patients and controls suggesting that trisomy of the partial transcript was not likely to be the mechanism underlying CMTX3 pathology (Brewer et al., 2016). When testing genes for DHMN1, the expression of the partial transcript *UBE3C* was higher in patients when compared to controls which may suggest a gene dosage mechanism for DHMN1 (unpublished data). Interestingly, the inserted sequence from chromosome 7q36.3 contains the fully intact *MNX1/HB9* gene along with associated promoter and flanking sequences. *MNX1/HB9* encodes a motor neuron transcription factor which is crucial for the consolidation of motor neuron identity (Arber et al., 1999). Models of ALS (Peviani et al., 2012) and CMT2A (Detmer, Vande Velde, Cleveland, & Chan, 2008) have used the *MNX1/HB9* gene promoter to express genes of interest in mouse motor neurons. Although a gene dosage effect cannot currently be ruled out for DHMN1, these studies suggest that it is highly plausible that the *MNX1/HB9* promoter or flanking regulatory sequences could be driving expression of nearby genes in DHMN1 motor neurons. To address this question and the relevance of gene expression changes in peripheral nerve of DHMN1 patients these findings will need to be reproduced in patient‐derived neural tissue.

### 3.2 Disruption to transcriptional regulation

Gene dysregulation through aberrant transcriptional regulation is not unprecedented as a disease mechanism in IPN as point mutations have previously been reported in noncoding regulatory sequences (Tomaselli et al., 2017). Point mutations in the 5′ untranslated region (UTR), 3′ UTR (Ionasescu, Searby, Ionasescu, Neuhaus, & Werner, 1996) and the neural‐specific promoter (P2) sequences of the *GJB1* gene (Houlden et al., 2004) have been reported to cause CMTX1. Similarly, point mutations in the 5′ UTR of the *SEPT9* (OMIM:*604061) gene cause hereditary neuralgic amyotrophy (HNA) (Kuhlenbaumer et al., 2005). These mutations highlight the importance of noncoding sequence mutations in IPN as well as the broader context of human disease.

Structural variations like those causing CMTX3 and DHMN1 may disrupt transcriptional regulation of one or more gene(s) by altering the cis‐acting regulatory element environments. The cis‐acting regulatory environment contains a number of different sequences or “regulatory elements” that bind *trans‐*acting regulatory proteins (transcription factor binding proteins‐TFBPs) and modulate the activity of genes and other transcribed regions of the genome at distances of up to 2–3 Mb. Large genomic rearrangements have been shown to disrupt noncoding, regulatory DNA sequences that structurally alter the regulatory environment. Significant changes in the activity of one or more genes resulting in disease can occur by disrupting the interaction between a gene(s) and regulatory sequences (such as promoters, enhancers/repressors) or introducing new or altered chromatin interactions (Dixon et al., 2012; Lupianez et al., 2015; Spielmann & Mundlos, 2013).

Quantitative gene expression analysis showed that the CMTX3 candidate gene *FGF13* (OMIM:*300070) had increased expression in patient lymphoblasts compared to controls and confirmed the complex insertion of chromosome 8q24.3 sequence into chromosome Xq27.1 can dysregulate genes in the CMTX3 linkage region. Further gene expression studies in disease‐relevant tissues for candidate genes mapping to the CMTX3 and DHMN1 loci are therefore necessary to fully elucidate the pathogenic consequences of the CMTX3 and DHMN1 complex insertions.

Interestingly, several other phenotypes in addition to CMTX3 have been reported in which different large chromosomal sequences have been inserted into the same region of chromosome Xq27.1. These include ptosis (Bunyan et al., 2014), hyperthyroidism (Bowl et al., 2005), hypertrichosis (De Stefano et al., 2013; Zhu et al., 2011), and XX male sex reversal (Haines et al., 2015). Although a precise disease mechanism is yet to be elucidated, the multiple phenotypes observed due to insertions at chromosome Xq27.1 raises two possibilities: (i) juxtaposition of a candidate gene with a regulatory element that would otherwise not normally regulate that gene; (ii) the inserted sequence could contain gene regulatory elements which, placed in proximity to genes in the CMTX3 linkage region may result in ectopic neural specific expression.

### 3.3 Altered chromatin environment

Another important consideration for pathogenic mechanisms underlying the CMTX3 and DHMN1 SV mutations is the effect of structural changes at the chromatin level of organization. Changes in the chromatin structure at a given locus can mediate the interaction between genes and their associated regulatory sequences (Kleinjan & Lettice, 2008; Schluth‐Bolard, Ottaviani, Gilson, & Magdiner, 2011). It is becoming increasingly recognized that these interactions take place within the context of a “3D genome” in which multiple, complex genome interactions, and configurations determine important biological functions such as DNA replication, DNA repair, and transcription (Bonev & Cavalli, 2016). Chromosome conformation capture studies examining the frequency of chromatin interactions have demonstrated that functions of the genome such as control of gene expression are moderated by chromosomes being linearly partitioned into topologically associated domains (TADs) (Bonev & Cavalli, 2016; Dixon et al., 2012; Krijger & De Laat, 2016; Olivares‐Chauvet et al., 2016). TADs are regions that demarcate chromosomal microenvironments in which sequences far apart in the genome preferentially come in close proximity to make contact with each other (Bouwman & De Laat, 2015). The contact between these linearly disparate sequences occurs through the 3D conformation of the genome. Little is known about the extent to which pathogenic SVs are known to alter the spatial organization of the genome causing disease. However, work in developmental and congenital disorders and cancer have provided some useful insights into the potential mechanisms that underpin the gene dysregulation resulting from altered spatial organization of the genome. These include (i) disruption to TAD boundaries, resulting in aberrant enhancer‐promoter interactions; (ii) altering the contents of TADs, thereby introducing novel interactions (reviewed in (Kaiser & Semple, 2017)). Interestingly, studies have shown that the architecture of TADs remains relatively conserved at the megabase scale between tissue types (Berlivet et al., 2013; Dixon et al., 2012). However, at the sub‐megabase scale there is evidence suggesting that “sub‐TADs” (Phillips‐Cremins et al., 2013) can vary among different tissues (Berlivet et al., 2013; Phillips‐Cremins et al., 2013), particularly for differentially expressed genes (Dixon et al., 2012). Therefore, it is plausible that CMTX3 and DHMN1 could represent ‘TADopathies’ (Matharu & Ahituv, 2015) in which the disruption of sub‐megabase scale interactions within peripheral nerve sub‐TADs could produce neuropathy. The CMTX3 and DHMN1 genomic rearrangements, therefore represent an ideal naturally occurring paradigm to study the disruption of chromatin organization and the impact on gene regulation.

## 4 CHALLENGES AND FUTURE STRATEGIES TO STUDY SV CAUSING IPNS

Unlike protein coding mutations, SV mutations such as those causing CMTX3 and DHMN1 may not immediately reveal a causative gene. This raises some important considerations and challenges within this exciting area of IPN research.

Large SVs causing pathogenic genomic rearrangements will increase the number of candidate genes to consider as causative. In some diseases, multiple genes disrupted by SV events may contribute to disease pathology (reviewed in (Iyer & Girirajan, 2015)). For CMT1A/HNPP, the 1.5 Mb region which encompasses 8 gene transcripts and several noncoding RNAs is specifically caused by trisomy of the *PMP22* gene. Given that CMTX3 and DHMN1 are Mendelian diseases, it is likely that the neuropathy will be caused by mutations affecting a single gene. In the event that multiple genes are dysregulated, identifying point mutations in these candidate genes that segregate in unsolved families could provide further evidence for the gene being causative. This was the case for CMT1A and HNPP where point mutations in the *PMP22* gene were identified in families lacking the 1.5 Mb duplication/deletion (Nicholson et al., 1994; Roa et al., 1993).

To assess the effects of SV mutations, appropriate models are required. Modelling large genomic rearrangements like the CMTX3 and DHMN1 complex insertions are difficult as they exceed the size limit and cloning accuracy of current technologies. In addition, obtaining disease relevant tissue is not possible prior to postmortem. Many studies for IPN have examined gene expression in alternative tissues such as lymphoblast (Zimon et al., 2012) and fibroblast cells (Echaniz‐Laguna et al., 2013; Kennerson et al., 2010). For CMTX3 and DHMN1, observable differences in the expression of *FGF13* and *UBE3C* have been identified in lymphoblasts. However, given that the regulation of gene transcription is likely to occur in a neural tissue specific manner it is not yet clear whether these observed differences are relevant to the CMTX3 and DHMN1 phenotype. To address examining gene expression in neural tissue, motor neurons derived from patient‐induced pluripotent stem cells (iPSCs) are now being used. Several studies have used iPSC‐derived motor neurons (iPSC‐MNs) to model neuromuscular diseases including ALS (Chen et al., 2014; Ichiyanagi et al., 2016; S. Lee & Huang, 2017), SMA (Fuller et al., 2015; Nizzardo et al., 2015) and various IPNs (G. Lee et al., 2009; Saporta et al., 2015). iPSCs‐MNs have the potential to model neural specific phenotypic changes, gene expression, and chromatin interactions in disease relevant tissue. This will help to elucidate the disease pathology and identify biological pathways to target for the development of appropriate therapies.

## 5 CONCLUSION

Inherited peripheral neuropathies and other Mendelian diseases have benefited from WES to interrogate protein coding regions of the genome. However, despite the advances in gene discovery and diagnostic testing many cases still remain unsolved. With WGS becoming more cost‐effective, and the improvements in technologies to generate longer sequencing reads, looking beyond the exome for SV mutations is feasible and an important consideration for unsolved cases of IPN and other Mendelian diseases. The current active research to understand the role of genomic interactions in gene regulation will be facilitated by the use of patient derived neural tissue from CMTX3 and DHMN1 patients through iPSC technologies. This review highlights the contribution of SV mutations and gene dysregulation as an important development in understanding the pathogenesis of IPNs.

## CONFLICT OF INTEREST

None declared.

## References

[mgg3390-bib-0001] Ainsworth, P. J. , Bolton, C. F. , Murphy, B. C. , Stuart, J. A. , & Hahn, A. F. (1998). Genotype/phenotype correlation in affected individuals of a family with a deletion of the entire coding sequence of the connexin 32 gene. Human Genetics, 103(2), 242–244. https://doi.org/10.1007/s004390050812 9760211 10.1007/s004390050812

[mgg3390-bib-0002] Alkan, C. , Coe, B. P. , & Eichler, E. E. (2011). Genome structural variation discovery and genotyping. Nature Reviews: Genetics, 12, 363–375. https://doi.org/10.1038/nrg2958 10.1038/nrg2958 PMC4108431 21358748

[mgg3390-bib-0003] Antonarakis, S. E. , Kazazian, H. H. , & Tuddenham, E. G. (1995). Molecular etiology of factor VIII deficiency in hemophilia A. Human Mutation, 5(1), 1–22. https://doi.org/10.1002/(ISSN)1098-1004 7728145 10.1002/humu.1380050102

[mgg3390-bib-0004] Arber, S. , Han, B. , Mendelsohn, M. , Smith, M. , Jessell, T. M. , & Sockanathan, S. (1999). Requirement for the homeobox gene Hb9 in the consolidation of motor neuron identity. Neuron, 23(4), 659–674. https://doi.org/10.1016/S0896-6273(01)80026-X 10482234 10.1016/s0896-6273(01)80026-x

[mgg3390-bib-0005] Baets, J. , & Timmerman, V. (2011). Inherited peripheral neuropathies: A myriad of genes and complex phenotypes. Brain, 134, 1585–1590. 21616967 10.1093/brain/awr114

[mgg3390-bib-0006] Baker, M. (2012). Structural variation: The Genome's hidden architechture. Nature Methods, 9(2), 133–137. https://doi.org/10.1038/nmeth.1858 22290183 10.1038/nmeth.1858

[mgg3390-bib-0007] Barber, K. E. , Ford, A. M. , Harris, R. L. , Harrison, C. J. , & Moorman, A. V. (2004). Mll translocations with concurrent 3' deletions: Interpretation of fish results. Genes Chromosomes Cancer, 41, 266–271. https://doi.org/10.1002/(ISSN)1098-2264 15334550 10.1002/gcc.20082

[mgg3390-bib-0008] Berlivet, S. , Paquette, D. , Dumouchel, A. , Langlais, D. , Dostie, J. , & Kmita, M. (2013). Clustering of tissue‐specific sub‐tads accompanies the regulation of hoxa genes in developing limbs. PLoS Genetics, 9(12), E1004018 https://doi.org/10.1371/Journal.Pgen.1004018 24385922 10.1371/journal.pgen.1004018 PMC3873244

[mgg3390-bib-0009] Bonev, B. , & Cavalli, G. (2016). Organization and function of the 3D genome. Nature Reviews Genetics, 17(11), 661–678. https://doi.org/10.1038/Nrg.2016.112 10.1038/nrg.2016.112 27739532

[mgg3390-bib-0010] Bouwman, B. A. , & De Laat, W. (2015). Getting the genome in shape: The formation of loops, domains and compartments. Genome Biology, 16, 154 https://doi.org/10.1186/S13059-015-0730-1 26257189 10.1186/s13059-015-0730-1 PMC4536798

[mgg3390-bib-0011] Bowl, M. R. , Nesbit, M. A. , Harding, B. , Levy, E. , Jefferson, A. , Volpi, E. , … Thakker, R. V. (2005). An interstitial deletion‐insertion involving chromosomes 2p25.3 and Xq27.1, near Sox3, causes X‐linked recessive hypoparathyroidism. Journal of Clinical Investigation, 115(10), 2822–2831. https://doi.org/10.1172/jci24156 16167084 10.1172/JCI24156 PMC1201662

[mgg3390-bib-0012] Brewer, M. H. , Chaudhry, R. , Qi, J. , Kidambi, A. , Drew, A. P. , Menezes, M. P. , … Kennerson, M. L. (2016). Whole genome sequencing identifies a 78 Kb insertion from chromosome 8 as the cause of charcot‐marie‐tooth neuropathy CMTX3. PLoS Genetics, 12(7), E1006177 https://doi.org/10.1371/Journal.Pgen.1006177 27438001 10.1371/journal.pgen.1006177 PMC4954712

[mgg3390-bib-0013] Bunyan, D. J. , Robinson, D. O. , Tyers, A. G. , Huang, S. , Maloney, V. K. , Grand, F. H. , … Mcmullan, T. F. W. (2014). X‐linked dominant congenital ptosis cosegregating with an interstitial insertion of a chromosome 1p21.3 fragment into a quasipalindromic sequence in Xq27.1. Open Journal Of Genetics, 04(06), 415–425. https://doi.org/10.4236/ojgen.2014.46039

[mgg3390-bib-0014] Carr, A. S. , Polke, J. M. , Wilson, J. , Pelayo‐Negro, A. L. , Laura, M. , Nanji, T. , … Reilly, M. M. (2015). Mfn2 deletion of exons 7 and 8: Founder mutation in the UK population. Journal of the Peripheral Nervous System, 20(2), 67–71. https://doi.org/10.1111/Jns.12117 26114802 10.1111/jns.12117

[mgg3390-bib-0015] Chance, P. F. , Alderson, M. K. , Leppig, K. A. , Lensch, M. W. , Matsunami, N. , Smith, B. , … Bird, T. D. (1993). DNA deletion associated with hereditary neuropathy with liability to pressure palsies. Cell, 72(1), 143–151. https://doi.org/10.1016/0092-8674(93)90058-X 8422677 10.1016/0092-8674(93)90058-x

[mgg3390-bib-0016] Chen, H. , Qian, K. , Du, Z. , Cao, J. , Petersen, A. , Liu, H. , … Zhang, S. C. (2014). Modeling ALS with iPSCS reveals that mutant SOD1 misregulates neurofilament balance in motor neurons. Cell Stem Cell, 14(6), 796–809. https://doi.org/10.1016/J.Stem.2014.02.004 24704493 10.1016/j.stem.2014.02.004 PMC4230530

[mgg3390-bib-0017] De Stefano, G. M. , Fantauzzo, K. A. , Petukhova, L. , Kurban, M. , Tadin‐Strapps, M. , Levy, B. , … Christiano, A. M. (2013). Position effect on FGF13 associated with X‐linked congenital generalized hypertrichosis. Proceedings of the National Academy of Sciences of the United States of America, 110(19), 7790–7795. https://doi.org/10.1073/Pnas.1216412110 23603273 10.1073/pnas.1216412110 PMC3651487

[mgg3390-bib-0018] Detmer, S. A. , Vande Velde, C. , Cleveland, D. W. , & Chan, D. C. (2008). Hindlimb gait defects due to motor axon loss and reduced distal muscles in a transgenic mouse model of charcot‐marie‐tooth type 2a. Human Molecular Genetics, 17(3), 367–375. https://doi.org/10.1093/Hmg/Ddm314 17959936 10.1093/hmg/ddm314

[mgg3390-bib-0019] Dixon, J. R. , Selvaraj, S. , Kim, A. , Li, Y. , Shen, Y. , Hu, M. , … Ren, B. (2012). Topological domains in mammalian genomes identified by analysis of chromatin interactions. Nature, 485, 376–380. https://doi.org/10.1038/nature11082 22495300 10.1038/nature11082 PMC3356448

[mgg3390-bib-0020] Drew, A. P. , Cutrupi, A. N. , Brewer, M. H. , Nicholson, G. A. , & Kennerson, M. L. (2016). A 1.35 Mb DNA fragment is inserted into the DHMN1 locus on chromosome 7q34‐Q36.2. Human Genetics, 135(11), 1269–1278. https://doi.org/10.1007/S00439-016-1720-4 27487800 10.1007/s00439-016-1720-4

[mgg3390-bib-0021] Drew, A. P. , Zhu, D. , Kidambi, A. , Ly, C. , Tey, S. , Brewer, M. H. , … Kennerson, M. L. (2015). Improved inherited peripheral neuropathy genetic diagnosis by whole‐exome sequencing. Molecular Genetics & Genomic Medicine, 3(2), 143–154. https://doi.org/10.1002/Mgg3.126 25802885 10.1002/mgg3.126 PMC4367087

[mgg3390-bib-0022] Echaniz‐Laguna, A. , Ghezzi, D. , Chassagne, M. , Mayencon, M. , Padet, S. , Melchionda, L. , … Mousson De Camaret, B. (2013). Surf1 deficiency causes demyelinating charcot‐marie‐tooth disease. Neurology, 81(17), 1523–1530. https://doi.org/10.1212/Wnl.0b013e3182a4a518 24027061 10.1212/WNL.0b013e3182a4a518 PMC3888171

[mgg3390-bib-0023] Fantes, J. , Breen, M. , Boyle, S. , Brown, J. , Fletcher, J. , Jones, S. , … Hanson, I. (1995). Aniridia‐associated cytogenetic rearrangements suggest that a position effect may cause the mutant phenotype. Human Molecular Genetics, 4(3), 415–422. https://doi.org/10.1093/hmg/4.3.415 7795596 10.1093/hmg/4.3.415

[mgg3390-bib-0024] Feuk, L. , Carson, A. R. , & Scherer, S. W. (2006). Structural variation in the human genome. Nature Reviews Genetics, 7, 85–97. https://doi.org/10.1038/nrg1767 10.1038/nrg1767 16418744

[mgg3390-bib-0025] Fukushima, Y. , Hoovers, J. , Mannens, M. , Wakui, K. , Ohashi, H. , Ohno, T. , … Niikawa, N. (1993). Detection of a cryptic paracentric inversion within band 11p13 in familial aniridia by fluorescence in situ hybridization. Human Genetics, 91, 205–209. 8478003 10.1007/BF00218257

[mgg3390-bib-0026] Fuller, H. R. , Mandefro, B. , Shirran, S. L. , Gross, A. R. , Kaus, A. S. , Botting, C. H. , … Sareen, D. (2015). Spinal muscular atrophy patient Ipsc‐derived motor neurons have reduced expression of proteins important in neuronal development. Frontiers in Cellular Neuroscience, 9, 506 https://doi.org/10.3389/Fncel.2015.00506 26793058 10.3389/fncel.2015.00506 PMC4707261

[mgg3390-bib-0027] Gopinath, S. , Blair, I. P. , Kennerson, M. , Jennifer, C. D. , & Nicholson, G. A. (2007). A novel locus for distal motor neuron degeneration maps to chromosome 7q34‐Q36. Human Genetics, 121(5), 559–564. https://doi.org/10.1007/s00439-007-0348-9 17354000 10.1007/s00439-007-0348-9

[mgg3390-bib-0028] Gu, W. , Zhang, F. , & Lupski, J. R. (2008). Mechanisms for human genomic rearrangements. Pathogenetics, 1(4), 1–17. 19014668 10.1186/1755-8417-1-4 PMC2583991

[mgg3390-bib-0029] Guan, P. , & Sung, W. K. (2016). Structural variation detection using next‐generation sequencing data: A comparative technical review. Methods, 102, 36–49. https://doi.org/10.1016/J.Ymeth.2016.01.020 26845461 10.1016/j.ymeth.2016.01.020

[mgg3390-bib-0030] Haines, B. , Hughes, J. , Corbett, M. , Shaw, M. , Innes, J. , Patel, L. , … Thomas, P. (2015). Interchromosomal insertional translocation at Xq26.3 alters SOX3 expression in an individual with XX male sex reversal. Journal of Clinical Endocrinology and Metabolism, 100(5), E815–E820. https://doi.org/10.1210/jc.2014-4383 25781358 10.1210/jc.2014-4383

[mgg3390-bib-0031] Hartley, T. , Wagner, J. D. , Warman‐Chardon, J. , Tetreault, M. , Brady, L. , Baker, S. , … Boycott, K. M. (2017). Whole‐exome sequencing is a valuable diagnostic tool for inherited peripheral neuropathies: Outcomes from A cohort of 50 families. Clinical Genetics, 93, 301–309. https://doi.org/10.1111/Cge.13101 28708278 10.1111/cge.13101

[mgg3390-bib-0032] Houlden, H. , Girard, M. , Cockerell, C. , Ingram, D. , Wood, N. W. , Goossens, M. , … Reilly, M. M. (2004). Connexin 32 promoter P2 mutations: A mechanism of peripheral nerve dysfunction. Annals of Neurology, 56(5), 730–734. https://doi.org/10.1002/Ana.20267 15470753 10.1002/ana.20267

[mgg3390-bib-0033] Hoyer, H. , Braathen, G. J. , Eek, A. K. , Nordang, G. B. , Skjelbred, C. F. , & Russell, M. B. (2015). Copy number variations in a population‐based study of Charcot‐Marie‐tooth disease. BioMed Research International, 2015, 960404 https://doi.org/10.1155/2015/960404 25648254 10.1155/2015/960404 PMC4306395

[mgg3390-bib-0034] Hoyer, H. , Braathen, G. J. , Eek, A. K. , Skjelbred, C. F. , & Russell, M. B. (2011). Charcot‐Marie‐Tooth caused by a copy number variation in myelin protein zero. European Journal of Medical Genetics, 54(6), E580–E583. https://doi.org/10.1016/J.Ejmg.2011.06.006 21787890 10.1016/j.ejmg.2011.06.006

[mgg3390-bib-0035] Hoyle, J. C. , Isfort, M. C. , Roggenbuck, J. , & Arnold, W. D. (2015). The genetics of Charcot‐Marie‐Tooth disease: Current trends and future implications for diagnosis and management. The Application of Clinical Genetics, 8, 235–243. https://doi.org/10.2147/Tacg.S69969 26527893 10.2147/TACG.S69969 PMC4621202

[mgg3390-bib-0036] Huang, J. , Wu, X. , Montenegro, G. , Price, J. , Wang, G. , Vance, J. M. , … Zuchner, S. (2010). Copy number variations are a rare cause of non‐CMT1A Charcot‐Marie‐Tooth disease. Journal of Neurology, 257(5), 735–741. https://doi.org/10.1007/S00415-009-5401-2 19949810 10.1007/s00415-009-5401-2 PMC2865568

[mgg3390-bib-0037] Hurles, M. E. , Dermitzakis, E. T. , & Tyler‐Smith, C. (2008). The functional impact of structural variation in humans. Trends in Genetics, 24(5), 238–245. https://doi.org/10.1016/J.Tig.2008.03.001 18378036 10.1016/j.tig.2008.03.001 PMC2869026

[mgg3390-bib-0038] Huttner, I. G. , Kennerson, M. L. , Reddel, S. W. , Radovanovic, D. , & Nicholson, G. A. (2006). Proof of genetic heterogeneity in X‐linked Charcot‐Marie‐Tooth disease. Neurology, 67(11), 2016–2021. https://doi.org/10.1212/01.wnl.0000247271.40782.b7 17159110 10.1212/01.wnl.0000247271.40782.b7

[mgg3390-bib-0039] Ichiyanagi, N. , Fujimori, K. , Yano, M. , Ishihara‐Fujisaki, C. , Sone, T. , Akiyama, T. , … Okano, H. (2016). Establishment of in vitro Fus‐associated familial amyotrophic lateral sclerosis model using human induced pluripotent stem cells. Stem Cell Reports, 6(4), 496–510. https://doi.org/10.1016/J.Stemcr.2016.02.011 26997647 10.1016/j.stemcr.2016.02.011 PMC4834049

[mgg3390-bib-0040] Inoue, K. , Dewar, K. , Katsanis, N. , Reiter, L. T. , Lander, E. S. , Devon, K. L. , … Birren, B. (2001). The 1.4mb CMT1A duplication/HNPP deletion genomic region reveals unique genome architechtural features and provides insights into the recent evoloution of new genes. Genome Research, 11, 1018–1033. https://doi.org/10.1101/gr.180401 11381029 10.1101/gr.180401 PMC311111

[mgg3390-bib-0041] Ionasescu, V. V. , Searby, C. , Ionasescu, R. , Neuhaus, I. M. , & Werner, R. (1996). Mutations of the noncoding region of the Connexin32 gene in X‐linked dominant Charcot‐Marie‐Tooth neuropathy. Neurology, 47(2), 541–544. https://doi.org/10.1212/WNL.47.2.541 8757034 10.1212/wnl.47.2.541

[mgg3390-bib-0042] Iyer, J. , & Girirajan, S. (2015). Gene discovery and functional assessment of rare copy‐number variants in neurodevelopmental disorders. Briefings in Functional Genomics, 14(5), 315–328. https://doi.org/10.1093/Bfgp/Elv018 25971441 10.1093/bfgp/elv018

[mgg3390-bib-0043] Jones, E. A. , Brewer, M. H. , Srinivasan, R. , Krueger, C. , Sun, G. , Charney, K. N. , … Svaren, J. (2012). Distal enhancers upstream of the Charcot‐Marie‐Tooth type 1a disease gene Pmp22. Human Molecular Genetics, 21(7), 1581–1591. https://doi.org/10.1093/Hmg/Ddr595 22180461 10.1093/hmg/ddr595 PMC3298281

[mgg3390-bib-0044] Jones, E. A. , Lopez‐Anido, C. , Srinivasan, R. , Krueger, C. , Chang, L. W. , Nagarajan, R. , & Svaren, J. (2011). Regulation of the PMP22 gene through an intronic enhancer. Journal of Neuroscience, 31(11), 4242–4250. https://doi.org/10.1523/Jneurosci.5893-10.2011 21411665 10.1523/JNEUROSCI.5893-10.2011 PMC3100536

[mgg3390-bib-0045] Kaiser, V. B. , & Semple, C. A. (2017). When tads go bad: Chromatin structure and nuclear organisation in human disease. F1000Research, 6, 1–8. https://doi.org/10.12688/f1000research.10792.1 10.12688/f1000research.10792.1 PMC5373421 28408976

[mgg3390-bib-0046] Katona, I. , Wu, X. , Feely, S. M. E. , Sottile, S. , Siskind, C. E. , Miller, L. J. , … Li, J. (2009). PMP22 expression in dermal nerve myelin from patients with CMT1A. Brain, 132, 1734–1740. https://doi.org/10.1093/brain/awp113 19447823 10.1093/brain/awp113 PMC2724915

[mgg3390-bib-0047] Kennerson, M. L. , Nicholson, G. A. , Kaler, S. G. , Kowalski, B. , Mercer, J. F. , Tang, J. , … Garbern, J. Y. (2010). Missense mutations in the copper transporter gene Atp7a cause X‐linked distal hereditary motor neuropathy. American Journal of Human Genetics, 86(3), 343–352. https://doi.org/10.1016/J.Ajhg.2010.01.027 20170900 10.1016/j.ajhg.2010.01.027 PMC2833394

[mgg3390-bib-0048] Kleinjan, D. A. , & Lettice, L. A. (2008). Long‐range gene control and genetic disease. Advances In Genetics, 61, 340–388. 10.1016/S0065-2660(07)00013-2 18282513

[mgg3390-bib-0049] Kleinjan, D. A. , & Van Heyningen, V. (2005). Long‐range control of gene expression: Emerging mechanisms and disruption in disease. American Journal Of Human Genetics, 76, 8–32. https://doi.org/10.1086/426833 15549674 10.1086/426833 PMC1196435

[mgg3390-bib-0050] Kostiner, D. R. , Nguyen, H. , Cox, V. A. , & Cotter, P. D. (2002). Stabilization of a terminal inversion duplication of 8p by telomere capture from 18q. Cytogenic Genome Research, 98, 9–12. https://doi.org/10.1159/000068536 10.1159/000068536 12584435

[mgg3390-bib-0051] Krijger, P. H. , & De Laat, W. (2016). Regulation of disease‐associated gene expression in the 3D genome. Nature Reviews Molecular Cell Biology, 17(12), 771–782. https://doi.org/10.1038/Nrm.2016.138 27826147 10.1038/nrm.2016.138

[mgg3390-bib-0052] Kuhlenbaumer, G. , Hannibal, M. C. , Nelis, E. , Schirmacher, A. , Verpoorten, N. , Meuleman, J. , … Chance, P. F. (2005). Mutations in SEPT9 cause hereditary neuralgic amyotrophy. Nature Genetics, 37(10), 1044–1046. https://doi.org/10.1038/Ng1649 16186812 10.1038/ng1649

[mgg3390-bib-0053] Lee, S. , & Huang, E. J. (2017). Modeling ALS and FTD with IPSC‐derived neurons. Brain Research, 1656, 88–97. https://doi.org/10.1016/J.Brainres.2015.10.003 26462653 10.1016/j.brainres.2015.10.003 PMC4833714

[mgg3390-bib-0054] Lee, G. , Papapetrou, E. P. , Kim, H. , Chambers, S. M. , Tomishima, M. J. , Fasano, C. A. , … Studer, L. (2009). Modelling pathogenesis and treatment of familial dysautonomia using patient‐specific Ipscs. Nature, 461(7262), 402–406. https://doi.org/10.1038/nature08320 19693009 10.1038/nature08320 PMC2784695

[mgg3390-bib-0055] Lin, C. , Numakura, C. , Ikegami, T. , Shizuka, M. , Shoji, M. , Nicholson, G. , & Hayasaka, K. (1999). Deletion and nonsense mutations of the connexin 32 gene associated with Charcot‐Marie‐Tooth disease. Tohoku Journal of Experimental Medicine, 188(3), 239–244. https://doi.org/10.1620/tjem.188.239 10587015 10.1620/tjem.188.239

[mgg3390-bib-0056] Lupianez, D. G. , Kraft, K. , Heinrich, V. , Krawitz, P. , Brancati, F. , Klopocki, E. , … Mundlos, S. (2015). Disruptions of topological chromatin domains cause pathogenic rewiring of gene‐enhancer interactions. Cell, 161(5), 1012–1025. https://doi.org/10.1016/J.Cell.2015.04.004 25959774 10.1016/j.cell.2015.04.004 PMC4791538

[mgg3390-bib-0057] Lupo, V. , Garcia‐Garcia, F. , Sancho, P. , Tello, C. , Garcia‐Romero, M. , Villarreal, L. , … Espinos, C. (2016). Assessment of targeted next‐generation sequencing as a tool for the diagnosis of Charcot‐Marie‐Tooth disease and hereditary motor neuropathy. The Journal of Molecular Diagnostics, 18(2), 225–234. https://doi.org/10.1016/J.Jmoldx.2015.10.005 26752306 10.1016/j.jmoldx.2015.10.005

[mgg3390-bib-0058] Lupski, J. R. , De Oca‐Luna, R. M. , Slaugenhaupt, S. , Pentao, L. , Guzzetta, V. , Trask, B. J. , … Patel, P. I. (1991). DNA duplication associated with Charcot‐Marie‐Tooth disease type 1a. Cell, 66(2), 219–232. https://doi.org/10.1016/0092-8674(91)90613-4 1677316 10.1016/0092-8674(91)90613-4

[mgg3390-bib-0059] Lupski, J. R. , Wise, C. A. , Kuwano, A. , Pentao, L. , Parke, J. T. , Glaze, D. G. , … Patel, P. I. (1992). Gene dosage is a mechanism for Charcot‐Marie‐Tooth disease type 1a. Nature Genetics, 1, 29–33. https://doi.org/10.1038/ng0492-29 1301995 10.1038/ng0492-29

[mgg3390-bib-0060] Maeda, M. H. , Mitsui, J. , Soong, B. W. , Takahashi, Y. , Ishiura, H. , Hayashi, S. , … Tsuji, S. (2012). Increased gene dosage of myelin protein zero causes Charcot‐Marie‐Tooth disease. Annals of Neurology, 71(1), 84–92. https://doi.org/10.1002/Ana.22658 22275255 10.1002/ana.22658

[mgg3390-bib-0061] Marongiu, R. , Brancati, F. , Antonini, A. , Ialongo, T. , Ceccarini, C. , Scarciolla, O. , … Valente, E. M. (2007). Whole gene deletion and splicing mutations expand the Pink1 genotypic spectrum. Human Mutation, 28(1), 98 https://doi.org/10.1002/Humu.9472 10.1002/humu.9472 17154281

[mgg3390-bib-0062] Matharu, N. , & Ahituv, N. (2015). Minor loops in major folds: Enhancer‐promoter looping, chromatin restructuring, and their association with transcriptional regulation and disease. PLoS Genetics, 11(12), E1005640 https://doi.org/10.1371/Journal.Pgen.1005640 26632825 10.1371/journal.pgen.1005640 PMC4669122

[mgg3390-bib-0063] Nadal, M. , Valiente, A. , Domenech, A. , Pritchard, M. , Estivill, X. , & Ramos‐Arroyo, M. A. (2000). Hereditary neuropathy with liability to pressure palsies: Two cases with a reciprocal translocation T(16;17)(Q12;11.2) interrupting the PMP22 gene. Journal of Medical Genetics, 37(5), 396–398. https://doi.org/10.1136/jmg.37.5.396 10905899 10.1136/jmg.37.5.396 PMC1734578

[mgg3390-bib-0064] Nakagawa, M. , Takashima, H. , Umehara, F. , Arimura, K. , Miyashita, F. , Takenouchi, N. , … Osame, M. (2001). Clinical phenotype in X‐linked Charcot‐Marie‐Tooth disease with an entire deletion of the connexin 32 coding sequence. Journal of the Neurological Sciences, 185(1), 31–37. https://doi.org/10.1016/S0022-510X(01)00454-3 11266688 10.1016/s0022-510x(01)00454-3

[mgg3390-bib-0065] Nicholson, G. A. , Valentijn, L. J. , Cherryson, A. K. , Kennerson, M. L. , Bragg, T. L. , Dekroon, R. M. , … Baas, F. (1994). A frame shift mutation in the PMP22 gene in hereditary neuropathy with liability to pressure palsies. Nature Genetics, 6(3), 263–266. https://doi.org/10.1038/Ng0394-263 8012388 10.1038/ng0394-263

[mgg3390-bib-0066] Nizzardo, M. , Simone, C. , Dametti, S. , Salani, S. , Ulzi, G. , Pagliarani, S. , … Corti, S. (2015). Spinal muscular atrophy phenotype is ameliorated in human motor neurons by Smn increase via different novel rna therapeutic approaches. Scientific Reports, 5, 11746 https://doi.org/10.1038/Srep11746 26123042 10.1038/srep11746 PMC4485234

[mgg3390-bib-0067] Okamoto, Y. , Goksungur, M. T. , Pehlivan, D. , Beck, C. R. , Gonzaga‐Jauregui, C. , Muzny, D. M. , … Lupski, J. R. (2014). Exonic duplication Cnv of NDRG1 associated with autosomal‐recessive HMSN‐Lom/CMT4D. Genetics in Medicine, 16(5), 386–394. https://doi.org/10.1038/Gim.2013.155 24136616 10.1038/gim.2013.155 PMC4224029

[mgg3390-bib-0068] Olivares‐Chauvet, P. , Mukamel, Z. , Lifshitz, A. , Schwartzman, O. , Elkayam, N. O. , Lubling, Y. , … Tanay, A. (2016). Capturing pairwise and multi‐way chromosomal conformations using chromosomal walks. Nature, 540(7632), 296–300. https://doi.org/10.1038/Nature20158 27919068 10.1038/nature20158

[mgg3390-bib-0069] Patel, P. I. , Roa, B. A. , Welcher, A. A. , Schoener‐Scott, R. , Trask, B. J. , Pentao, L. , … Suter, U. (1992). The gene for the peripheral myelin protein PMP‐22 is a candidate for Charcot‐Marie‐Tooth disease type 1a. Nature Genetics, 1, 159–165. https://doi.org/10.1038/ng0692-159 1303228 10.1038/ng0692-159

[mgg3390-bib-0070] Pehlivan, D. , Beck, C. R. , Okamoto, Y. , Harel, T. , Akdemir, Z. H. , Jhangiani, S. N. , … Lupski, J. R. (2016). The role of combined SNV and CNV burden in patients with distal symmetric polyneuropathy. Genetics in Medicine, 18(5), 443–451. https://doi.org/10.1038/Gim.2015.124 26378787 10.1038/gim.2015.124 PMC5322766

[mgg3390-bib-0071] Peviani, M. , Kurosaki, M. , Terao, M. , Lidonnici, D. , Gensano, F. , Battaglia, E. , … Bendotti, C. (2012). Lentiviral vectors carrying enhancer elements of Hb9 promoter drive selective transgene expression in mouse spinal cord motor neurons. Journal of Neuroscience Methods, 205(1), 139–147. https://doi.org/10.1016/J.Jneumeth.2011.12.024 22245491 10.1016/j.jneumeth.2011.12.024

[mgg3390-bib-0072] Phillips‐Cremins, J. E. , Sauria, M. E. , Sanyal, A. , Gerasimova, T. I. , Lajoie, B. R. , Bell, J. S. , … Corces, V. G. (2013). Architectural protein subclasses shape 3D organization of genomes during lineage commitment. Cell, 153(6), 1281–1295. https://doi.org/10.1016/J.Cell.2013.04.053 23706625 10.1016/j.cell.2013.04.053 PMC3712340

[mgg3390-bib-0073] Redon, R. , Ishikawa, S. , Fitch, K. R. , Feuk, L. , Perry, G. H. , Andrews, T. D. , … Hurles, M. E. (2006). Global variation in copy number in the human genome. Nature, 444(7118), 444–454. https://doi.org/10.1038/Nature05329 17122850 10.1038/nature05329 PMC2669898

[mgg3390-bib-0074] Roa, B. B. , Garcia, C. A. , Suter, U. , Kulpa, D. A. , Wise, C. A. , Mueller, J. , … Lupski, J. R. (1993). Charcot‐Marie‐Tooth disease type 1a – association with a spontaneous point mutation in the PMP22 gene. New England Journal Of Medicine, 329(2), 96–101. https://doi.org/10.1056/Nejm199307083290205 8510709 10.1056/NEJM199307083290205

[mgg3390-bib-0075] Rouger, H. , Leguern, E. , Birouk, N. , Gouider, R. , Tardieu, S. , Plassart, E. , … Brice, A. (1997). Charcot‐Marie‐Tooth disease with intermediate motor nerve conduction velocities: Characterization of 14 Cx32 mutations in 35 families. Human Mutation, 10(6), 443–452. https://doi.org/10.1002/(Sici)1098-1004(1997)10:6<443:Aid-Humu5>3.0.Co;2-E 9401007 10.1002/(SICI)1098-1004(1997)10:6<443::AID-HUMU5>3.0.CO;2-E

[mgg3390-bib-0076] Saporta, M. A. , Dang, V. , Volfson, D. , Zou, B. , Xie, X. S. , Adebola, A. , … Dimos, J. T. (2015). Axonal Charcot‐Marie‐Tooth disease patient‐derived motor neurons demonstrate disease‐specific phenotypes including abnormal electrophysiological properties. Experimental Neurology, 263, 190–199. https://doi.org/10.1016/J.Expneurol.2014.10.005 25448007 10.1016/j.expneurol.2014.10.005 PMC4262589

[mgg3390-bib-0077] Schabhuttl, M. , Wieland, T. , Senderek, J. , Baets, J. , Timmerman, V. , De Jonghe, P. , … Auer‐Grumbach, M. (2014). Whole‐exome sequencing in patients with inherited neuropathies: Outcome and challenges. Journal of Neurology, 261(5), 970–982. https://doi.org/10.1007/S00415-014-7289-8 24627108 10.1007/s00415-014-7289-8

[mgg3390-bib-0078] Schluth‐Bolard, C. , Ottaviani, A. , Gilson, E. , & Magdiner, F. (2011). Chromosomal Position Effects And Gene Variegation: Impact In Pathologies In Tollefsbol T. (Ed.), Handbook Of Epigenetics: The New Molecular And Medical Genetics. Burlington, MA: Academic Press.

[mgg3390-bib-0079] Simola, K. O. , Knuutila, S. , Kaitila, I. , Pirkola, A. , & Pohja, P. (1983). Familial Aniridia and translocation T(4;11)(Q22;P13) without Wilms' tumor. Human Genetics, 63, 158–161. https://doi.org/10.1007/BF00291536 6301974 10.1007/BF00291536

[mgg3390-bib-0080] Singleton, A. B. , Farrer, M. , Johnson, J. , Singleton, A. , Hague, S. , Kachergus, J. , Hulihan, M. , … Gwinn‐Hardy, K. (2003). Alpha‐synuclein locus triplication causes Parkinson's disease. Science, 302(5646), 841 https://doi.org/10.1126/science.1090278 14593171 10.1126/science.1090278

[mgg3390-bib-0081] Skre, H. (1974). Genetic and clinical aspects of charcot‐marie‐tooth's disease. Clinical Genetics, 6(2), 98–118. 4430158 10.1111/j.1399-0004.1974.tb00638.x

[mgg3390-bib-0082] Spielmann, M. , & Mundlos, S. (2013). Structural variations, the regulatory landscape of the genome and their alteration in human disease. BioEssays, 35(6), 533–543. https://doi.org/10.1002/Bies.201200178 23625790 10.1002/bies.201200178

[mgg3390-bib-0083] Stankiewicz, P. , & Lupski, J. R. (2010). Structural variation in the human genome and its role in disease. Annual Review Of Medicine, 61, 437–455. https://doi.org/10.1146/annurev-med-100708-204735 10.1146/annurev-med-100708-204735 20059347

[mgg3390-bib-0084] Timmerman, V. , Nelis, E. , Van Hul, W. , Nieuwenhuijsen, B. W. , Chen, K. L. , Wang, S. , Van Broeckhoven, C. (1992). The peripheral myelin protein gene Pmp‐22 is contained within the Charcot‐Marie‐Tooth disease type 1a duplication. Nature Genetics, 1, 171–175. https://doi.org/10.1038/ng0692-171 1303230 10.1038/ng0692-171

[mgg3390-bib-0085] Timmerman, V. , Strickland, A. V. , & Zuchner, S. (2014). Genetics of Charcot‐Marie‐Tooth (CMT) disease within the frame of the human genome project success. Genes (Basel), 5(1), 13–32. https://doi.org/10.3390/Genes5010013 24705285 10.3390/genes5010013 PMC3978509

[mgg3390-bib-0086] Tomaselli, P. J. , Rossor, A. M. , Horga, A. , Jaunmuktane, Z. , Carr, A. , Saveri, P. , … Reilly, M. M. (2017). Mutations in noncoding regions of Gjb1 are a major cause of X‐linked CMT. Neurology, 88, 1445–1453. https://doi.org/10.1212/WNL.0000000000003819 28283593 10.1212/WNL.0000000000003819 PMC5386440

[mgg3390-bib-0087] Tuddenham, E. G. D. , Cooper, D. N. , Gitschier, J. , Higuchi, M. , Hoyer, L. W. , Yoshioka, A. , … Antonarkis, S. E. (1991). Haemophilia A: Database of nucleotide substitutions, deletions, insertions and rearrangements of the factor VIII gene. Nucleic Acids Research, 19(18), 4821–4833. https://doi.org/10.1093/nar/19.18.4821 1923751 10.1093/nar/19.18.4821 PMC328775

[mgg3390-bib-0088] Valentijn, L. J. , Baas, F. , Zorn, I. , Hensels, G. W. , De Visser, M. , & Bolhuis, P. A. (1993). Alternatively sized duplication in Charcot‐Marie‐Tooth disease type 1a. Human Molecular Genetics, 2(12), 2143–2146. https://doi.org/10.1093/hmg/2.12.2143 8111385 10.1093/hmg/2.12.2143

[mgg3390-bib-0089] Valentijn, L. J. , Bolhuis, P. A. , Zorn, I. , Hoogendijk, J. E. , Van Den Bosch, N. , Hensels, G. W. , … Baas, F. (1992). The peripheral myelin gene PMP−22/GAS−3 is duplicated in Charcot−Marie−Tooth disease type 1a. Nature Genetics, 1, 166–170. https://doi.org/10.1038/ng0692-166 1303229 10.1038/ng0692-166

[mgg3390-bib-0090] Weischenfeldt, J. , Symmons, O. , Spitz, F. , & Korbel, J. O. (2013). Phenotypic impact of genomic structural variation: Insights from and for human disease. Nature Reviews Genetics, 14, 125–139. https://doi.org/10.1038/nrg3373 10.1038/nrg3373 23329113

[mgg3390-bib-0091] Weterman, M. A. , Van Ruissen, F. , De Wissel, M. , Bordewijk, L. , Samijn, J. P. , Van Der Pol, W. L. , … Baas, F. (2010). Copy number variation upstream of PMP22 in Charcot‐Marie‐Tooth disease. European Journal of Human Genetics, 18(4), 421–428. https://doi.org/10.1038/Ejhg.2009.186 19888301 10.1038/ejhg.2009.186 PMC2987248

[mgg3390-bib-0092] Zhang, F. , Seeman, P. , Liu, P. , Weterman, M. A. , Gonzaga‐Jauregui, C. , Towne, C. F. , … Lupski, J. R. (2010). Mechanisms for nonrecurrent genomic rearrangements associated with CMT1A or HNPP: Rare CNVs as a cause for missing heritability. American Journal of Human Genetics, 86(6), 892–903. https://doi.org/10.1016/J.Ajhg.2010.05.001 20493460 10.1016/j.ajhg.2010.05.001 PMC3032071

[mgg3390-bib-0093] Zhu, H. , Shang, D. , Sun, M. , Choi, S. , Liu, Q. , Hao, J. , … Zhang, X. (2011). X‐linked congenital hypertrichosis syndrome is associated with interchromosomal insertions mediated by a human‐specific palindrome near Sox3. American Journal of Human Genetics, 88(6), 819–826. https://doi.org/10.1016/J.Ajhg.2011.05.004 21636067 10.1016/j.ajhg.2011.05.004 PMC3113246

[mgg3390-bib-0094] Zimon, M. , Baets, J. , Almeida‐Souza, L. , De Vriendt, E. , Nikodinovic, J. , Parman, Y. , … Jordanova, A. (2012). Loss‐of‐function mutations in Hint1 cause axonal neuropathy with neuromyotonia. Nature Genetics, 44(10), 1080–1083. https://doi.org/10.1038/Ng.2406 22961002 10.1038/ng.2406

